# Supplementary material for: Lipoprotein lipase regulates hematopoietic stem progenitor cell maintenance through DHA supply
Source: Nat Commun. 2018 Apr 3;9:1310. doi: 10.1038/s41467-018-03775-y (PMC5882990; doi:10.1038/s41467-018-03775-y)
Supplement: Supplementary file 1 — Supplementary Information [file 41467_2018_3775_MOESM1_ESM.pdf]

Liu et al. Lipoprotein lipase regulates hematopoietic stem progenitor cell maintenance through docosahexaenoic acid supply.

Supplementary Information Figures

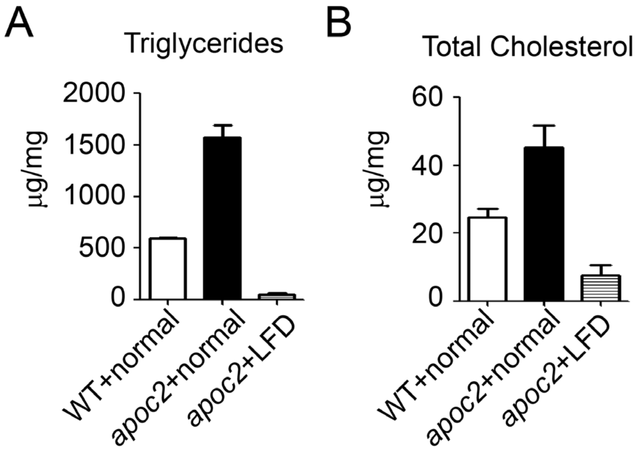

Supplementary Figure 1. Feeding a low-fat diet reduces hyperlipidemia in *apoc2* mutants.
Triglycerides (A) and total cholesterol (B) levels were measured in homogenates of 14 dpf larvae and normalized to total protein. Feeding normal or low-fat diet (LFD) started on 5th dpf. Triglycerides and total cholesterol were measured in a homogenate of 5 pooled larvae in each group. Mean±SD; n=3 (technical replicates).

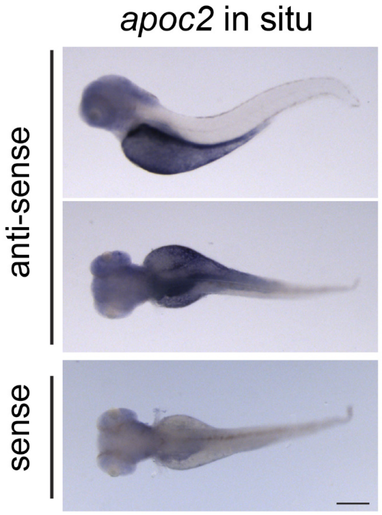

Supplementary Figure 2. *In situ* hybridization with *apoc2* antisense and sense probes.
In 2 dpf zebrafish, *apoc2* is mainly expressed in the intestine and the yolk.

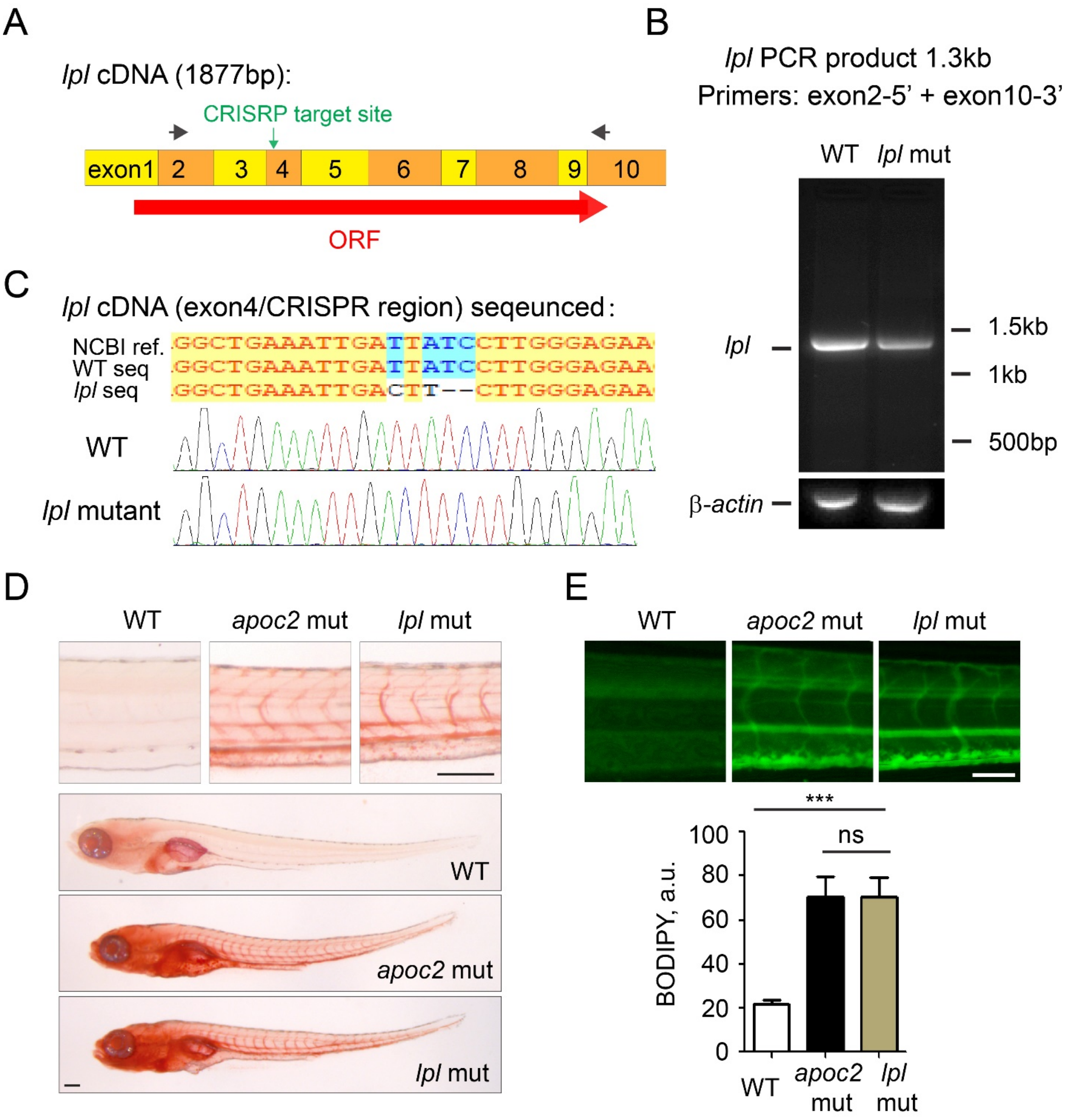

Supplementary Figure 3. *lpl* mutants develop hyperlipidemia. (A) Primers, flanking exon 4, the site of CRISPR target, are used to amplify *lpl* cDNA. (B) Single *lpl* cDNA band of the same size in WT and *lpl* mutant embryos indicates that no alternative splicing occurs in the mutant. (C) Sequence results indicate a 2nt deletion in cDNA from *lpl* mutants. (D) ORO staining of WT, *apoc2* and *lpl* mutants at 6 dpf. (E) Representative images and quantitative results of BODIPY neutral lipid staining of WT, *apoc2* and *lpl* mutants at 6 dpf. Results are mean±SEM; n=5 (WT and *apoc2* mut) and n=6 (*lpl* mut); ****P*<0.001 (Student's *t*-test). Scale bars, 100 µm in D, and 50 µm in E.

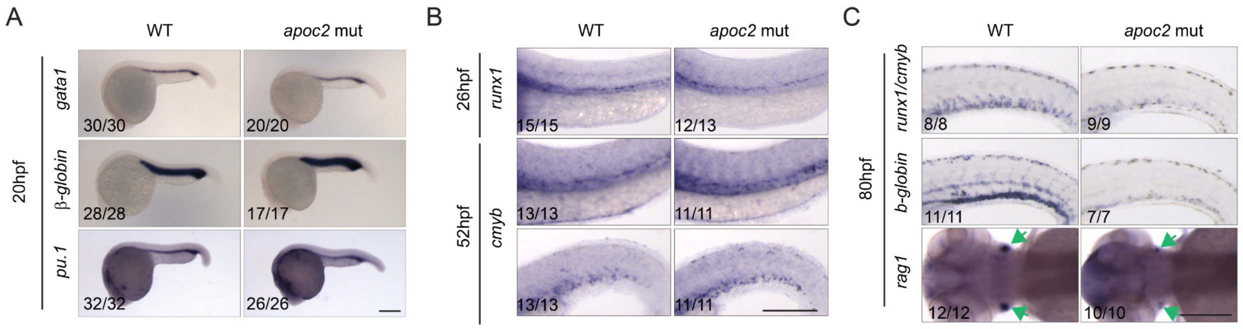

Supplementary Figure 4. Expression of hematopoietic markers in *apoc2* mutants at different developmental stages. (A) *In situ* hybridization with *gata1, β-globin* and *pu.1* probes in WT and *apoc2* mutants at 20 hpf. (B) *In situ* hybridization with *runx1* at 26 hpf (top) and *cmyb* at 52 hpf in WT and *apoc2* mutants' VDA (middle images) and CHT (bottom) regions. (C) *In situ* hybridization with *cmyb/runx1, β-globin* and *rag1* probes in WT and *apoc2* mutants at 80 hpf. *rag1* signals in the thymus are indicated with green arrows. Scale bars, 200 μm.

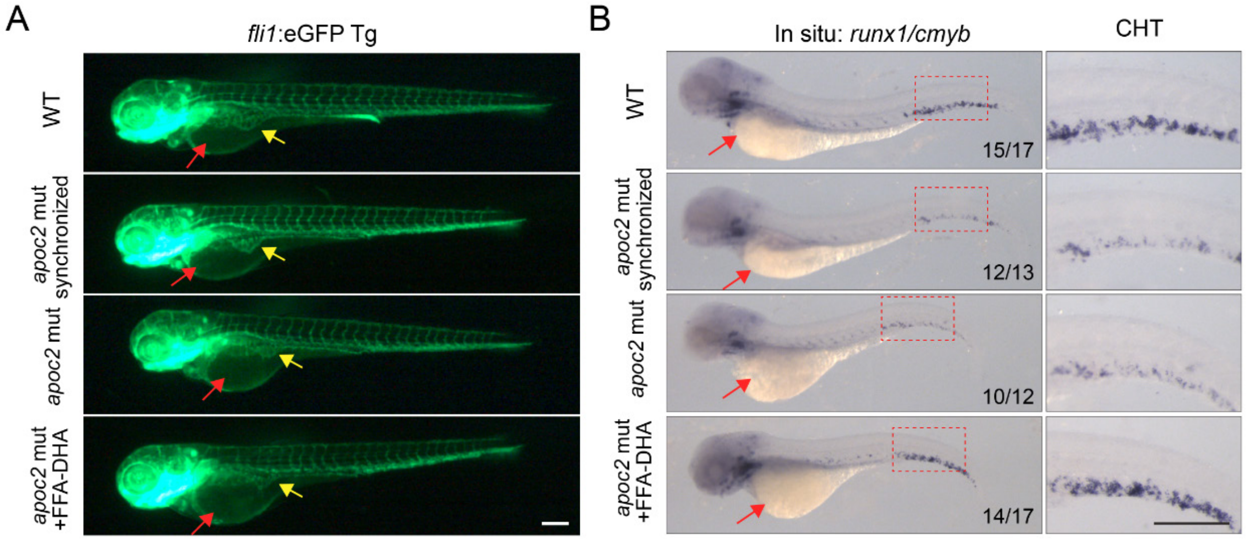

Supplementary Figure 5. Hematopoietic defect in *apoc2* mutants is not due to delayed angiogenesis. (A) Angiogenesis in *fli1:eGFP* WT, *apoc2* mutant, the *apoc2* mutant synchronized with WT, and the *apoc2* mutant injected with DHA, all imaged at 80 hpf. *apoc2* mutant synchronization was achieved by maintaining the embryos at 30°C from 72 to 80 hpf, while WT embryos were kept at 28°C. DHA was injected at 48 hpf. Yellow arrows point to sub-intestinal vessels. Red arrows point to the yolk. (B) *In situ* hybridization with *runx1/cmyb* primers in the same groups as in panel A, at 80 hpf. The CHT regions traced with red dashed quadrangles are enlarged in right-hand panels. Scale bars, 200 μm.

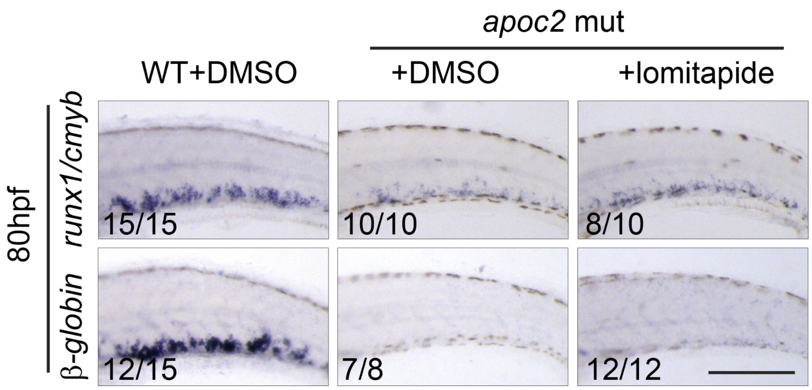

Supplementary Figure 6. Lomitapide does not rescue the hematopoietic defect in *apoc2* mutants. *In situ* hybridization with *cmyb/runx1* and *β-globin* probes in WT, *apoc2* mutants and the *apoc2* mutants treated with 5 µM lomitapide. Lomitapide treatment was started at 2 dpf and continued until the embryos were fixed at 80 hpf for *in situ* hybridization. Scale bar, 200 µm.

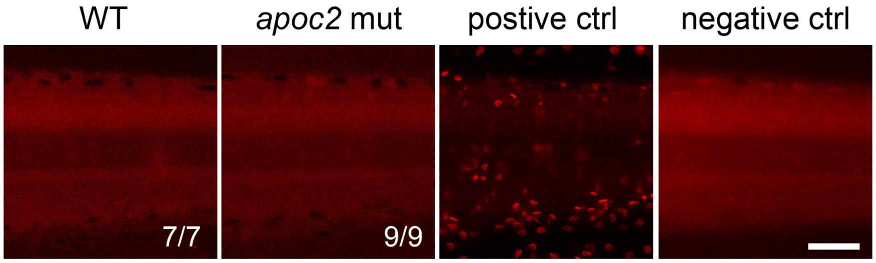

Supplementary Figure 7. *apoc2* mutants show no increase in apoptosis in the CHT region. TUNEL staining in the CHT region of 3.3 dpf WT and *apoc2* mutant embryos. Embryos incubated with Dnase I for 15 min at room temperature were used as a positive control. Embryos incubated with a labeling solution only, without terminal transferase, were used as a negative control. Scale bar, 50 µm.

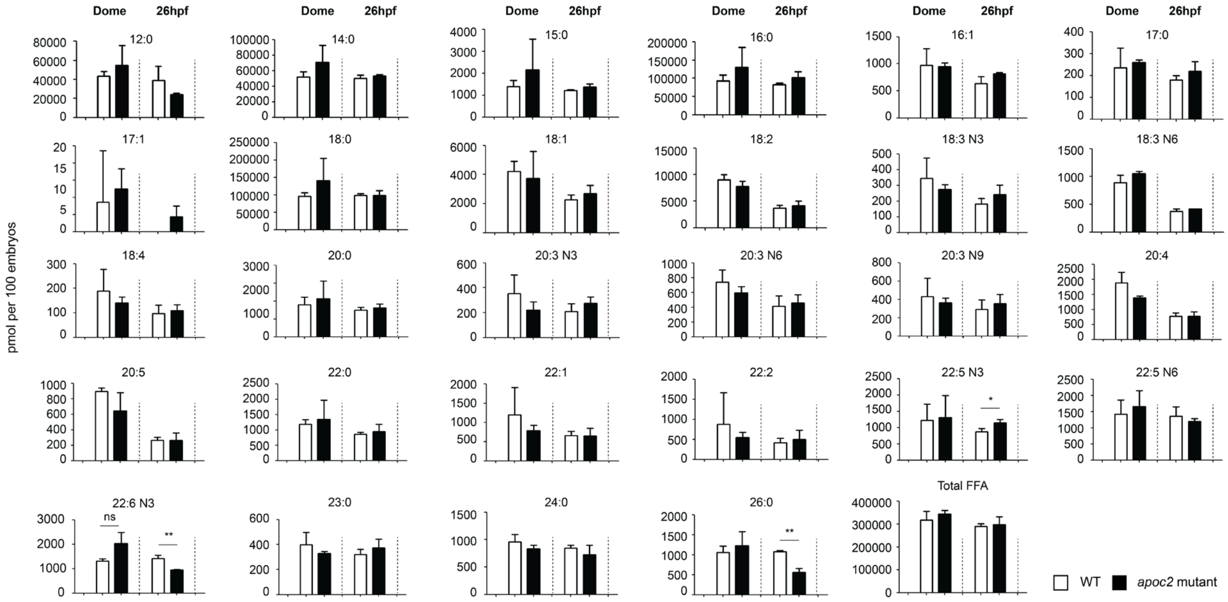

Supplementary Figure 8. GC-MS analysis of FFAs in zebrafish homogenates. Each replicate is a pool of 20 embryos. Results are mean±SEM; n=3 in each group, * P<0.05 and ** P<0.01 (Student's t-test).

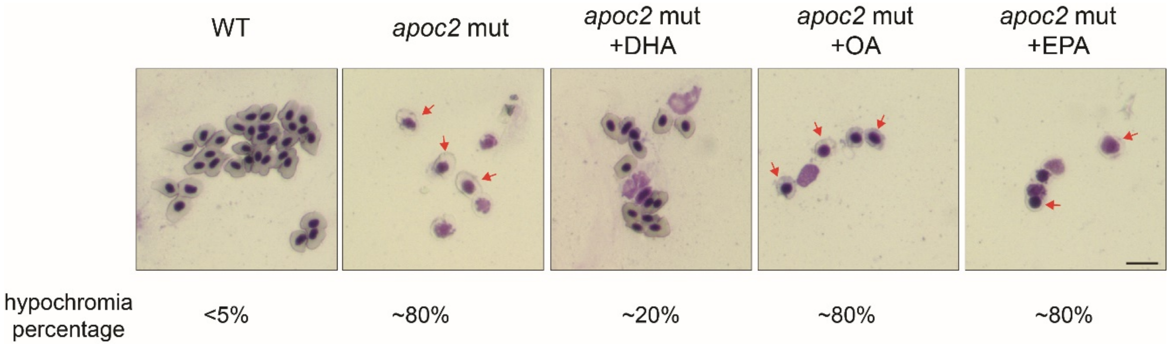

Supplementary Figure 9. DHA but not oleic or eicosapentaenoic acid rescues hypochromia in *apoc2* mutants. (A) *apoc2* mutant embryos were injected with free fatty acid DHA, OA or EPA at 48 hpf. Wright-Giemsa staining of peripheral blood cells in 6.3 dpf zebrafish. Red arrows point to immature erythrocytes containing larger and less condensed nuclei. Scale bar, 10 µm.

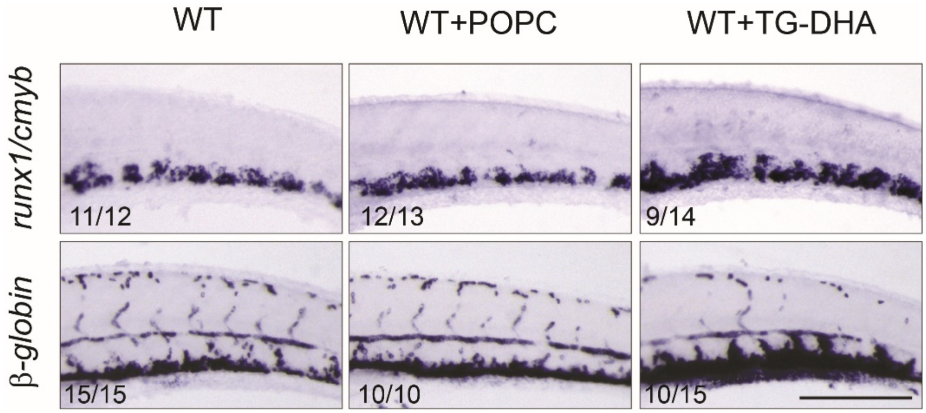

Supplementary Figure 10. TG-DHA but not the vehicle increases hematopoietic markers in WT zebrafish. *In situ* hybridization with *cmyb/runx1* and *β-globin* probes in WT and WT injected with POPC or TG-DHA:POPC micelles. Embryos were injected at 2 dpf and fixed at 80 hpf for *in situ* hybridization. Left- and right-hand side images are the same as in Fug. 8B. Images in the middle were obtained from the same experiment as those shown in Fig. 8B. Scale bar, 200 μm.

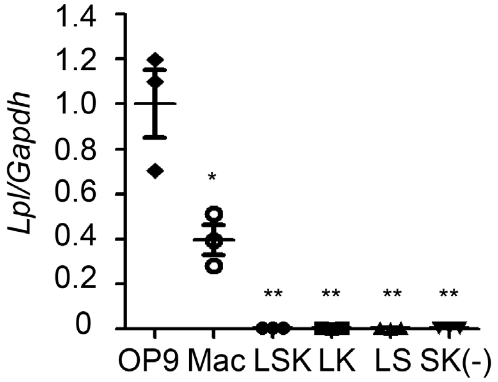

Supplementary Figure 11. *lpl* expression in murine OP9 stromal cells, macrophages (Mac) and lineage negative cells. LSK (Lineage negative, Sca1 and c-Kit positive), LK, LS and SK(-) cells were isolated from bone marrow of C57Bl6 mice. Results are mean±SEM; n=3 in each group, * $P<0.05$ and ** $P<0.01$ (Student's *t*-test).
