# Supplementary material for: Lipoprotein lipase regulates hematopoietic stem progenitor cell maintenance through DHA supply
Source: Nat Commun. 2018 Apr 3;9:1310. doi: 10.1038/s41467-018-03775-y (PMC5882990; doi:10.1038/s41467-018-03775-y)
Supplement: Supplementary file 3 — Description of Additional Supplementary Files [file 41467_2018_3775_MOESM3_ESM.pdf]

Description of Additional Supplementary Files

File Name: Supplementary Movie 1
Description: **Circulating blood cells in a 6.3 dpf wild type zebrafish larva.** Corresponding still image is in a bottom-left panel of Fig. 1C.

File Name: Supplementary Movie 2
Description: **Circulating blood cells in a 6.3 dpf apoc2 mutant zebrafish larva.** Corresponding still image is in a bottom-right panel of Fig. 1C.
